# Uric acid, lung function, physical capacity and exacerbation frequency in patients with COPD: a multi-dimensional approach

**DOI:** 10.1186/s12931-018-0815-y

**Published:** 2018-06-04

**Authors:** Kathrin Kahnert, Peter Alter, Tobias Welte, Rudolf M. Huber, Jürgen Behr, Frank Biertz, Henrik Watz, Robert Bals, Claus F. Vogelmeier, Rudolf A. Jörres

**Affiliations:** 1 Department of Internal Medicine V, University of Munich (LMU), Comprehensive Pneumology Center,Member of the German Center for Lung Research (DZL), Munich, Germany; 2 Department of Medicine, Pulmonary and Critical Care Medicine, University Medical Center Giessen and Marburg, Philipps-University Marburg, Germany, Member of the German Center for Lung Research (DZL), Baldingerstrasse, 35043 Marburg, Germany; 3 Department of Pneumology, Hannover Medical School, Carl-Neuberg-Str. 1, 30625 Hannover, Germany; 4 Institute for Biostatistics, Hannover Medical School, Carl-Neuberg-Str. 1, 30625 Hannover, Germany; 5 Pulmonary Research Institute at LungenClinic Grosshansdorf, Airway Research Center North, Member of the German Center for Lung Research, Woehrendamm 80, 22927 Grosshansdorf, Germany; 6 Department of Internal Medicine V – Pulmonology, Allergology, Respiratory Intensive Care Medicine, Saarland University Hospital, Kirrberger Straße 1, 66424 Homburg, Germany; 7 Institute and Outpatient Clinic for Occupational, Social and Environmental Medicine, Comprehensive Pneumology Center Munich (CPC-M), Ludwig-Maximilians-Universität München, Ziemssenstr. 1, 80336 Munich, Germany

**Keywords:** Uric acid, Comorbidity, COPD, Physical capacity, Exacerbations

## Abstract

**Background:**

Recent investigations showed single associations between uric acid levels, functional parameters, exacerbations and mortality in COPD patients. The aim of this study was to describe the role of uric acid within the network of multiple relationships between function, exacerbation and comorbidities.

**Methods:**

We used baseline data from the German COPD cohort COSYCONET which were evaluated by standard multiple regression analyses as well as path analysis to quantify the network of relations between parameters, particularly uric acid.

**Results:**

Data from 1966 patients were analyzed. Uric acid was significantly associated with reduced FEV_1_, reduced 6-MWD, higher burden of exacerbations (GOLD criteria) and cardiovascular comorbidities, in addition to risk factors such as BMI and packyears. These associations remained significant after taking into account their multiple interdependences. Compared to uric acid levels the diagnosis of hyperuricemia and its medication played a minor role.

**Conclusion:**

Within the limits of a cross-sectional approach, our results strongly suggest that uric acid is a biomarker of high impact in COPD and plays a genuine role for relevant outcomes such as physical capacity and exacerbations. These findings suggest that more attention should be paid to uric acid in the evaluation of COPD disease status.

## Background

Recent investigations showed an increased mortality in COPD patients with elevated uric acid (UA) levels [1, 2] and described UA as an independent predictor of 30-day mortality of acute exacerbations [2]. UA is known to be associated with markers of systemic inflammation [3], bronchoconstriction by stimulation of endothelin-1 [4, 5], as well as oxygen desaturation [6].Both lower and higher levels of UA have been described as risk factors for airway obstruction [7, 8], also in addition to restrictive pattern linked to UA [9]. Therefor the worse outcome of COPD patients with hyperuricemia seems to involve a number of factors including systemic inflammation, oxygen desaturation and lung function alterations. Hyperuricemia is also associated with an increasing risk of coronary heart disease [10], a comorbidity that is relevant for mortality in COPD patients [11]. As an overall marker of functional capacity in COPD the 6-min walk distance (6-MWD) is well established and known to be a stronger predictor of mortality than other markers of severe COPD [12]. In view of the complexity of the disease and interdependence of parameters, the functional changes related to uric acid [2, 13] may well include changes in 6-MWD, in addition to associations with comorbidities and exacerbations, even if common risk factors such as age, gender, smoking and body-mass index (BMI) have been taken into account. However, the multiple associations between parameters may render it difficult to quantify the causal role of UA and to separate direct and indirect effects from each other. This can be done using path analysis as a tool to integrate and cross-check the results of conventional regression analyses that have been performed for single outcome measures but never put into a comprehensive picture.

Based on this the aim of this study was to identify the role of the biomarker UA for spirometric parameters, 6-min walk distance, exacerbation rate and cardiovascular comorbidities in COPD while taking into account the fact that these outcome measures are related to each other. The hypothesis was that UA has direct effects on these measures that cannot be explained by their mutual relationships and common risk factors. Such information could be helpful to understand pathophysiological mechanisms and the responses to therapy. For this purpose we used data from the German COPD cohort study COSYCONET (**CO**PD and **Sy**stemic **Co**nsequences-Comorbidities **Net**work).

## Methods

### Study population

The analysis was based on the baseline data set of COSYCONET, which is a multi-center study with focus on comorbidities in COPD [14]. Patients were enrolled in COSYCONET, if the following inclusion criteria were fulfilled: aged 40 years and older, diagnosis of COPD or chronic bronchitis, availability for repeated study visits over at least 18 months and if none of the following exclusion criteria was fulfilled: having undergone major lung surgery (e.g. lung volume reduction, lung transplant), moderate or severe exacerbation within 4 weeks prior to the visit, having a lung tumor, physical or cognitive impairment resulting in an inability to walk or to understand the intention of the project [14].

From *n* = 2741patients recruited into COSYCONET only patients with complete data allowing the categorization into GOLD grades 1–4 and GOLD groups A-D (2017) [15] based on the COPD Assessment Test (CAT, threshold 10 according to GOLD recommendations [15]) were included into this analysis; this required valid values of forced expiratory volume in 1 s (FEV_1_), forced vital capacity (FVC) and CAT. Moreover, valid data on body-mass index (BMI), packyears of smoking, 6-min walk distance (6-MWD), serum levels of UA and creatinine, as well as hyperuricemia-related medication were required. The exacerbation risk for the ABCD grouping was based on the 12-month history of exacerbations of all severities, including hospitalization, as described by GOLD (with high risk indicated by a history of two non-hospitalized exacerbations, or one exacerbation leading to hospital admission) [15]. This resulted in a subset of 1966 out of 2741 patients recruited into COSYCONET [14]. The COSYCONET study had been approved by the ethical committees of all study centers, and all patients gave their written informed consent.

### Assessments

The study protocol and panel of assessments have been described previously [14]. The diagnosis of hyperuricemia used for the description of the baseline characteristics of the study cohort was based on patients’ reports of a doctor-based diagnosis, irrespective of medication. In the absence of a report, the diagnosis was also assumed if hyperuricemia-specific medication was identified; details of this procedure have been given previously [16]. In the statistical analyses we primarily included all patients, i.e. (a) patients without any diagnosis of hyperuricemia according to these criteria, (b) patients with the reported diagnosis plus hyperuricemia-specific medication, and (c) patients with the reported diagnosis but no hyperuricemia-specific medication. It should be acknowledged that in all analyses the target variable was UA and not the diagnosis of hyperuricemia. The diagnosis was used only in sensitivity analyses in which either group (b) or (c) were excluded to address of role of diagnosis and medication for the associations with UA. In addition to UA, we included the risk factors age, BMI, gender, smoking in terms of packyears, the functional parameters 6-min walk distance (6 MWD), FEV_1_ and FVC, each in % predicted, as well as a binary exacerbation category. The categorization of low and high exacerbations was based on the GOLD groups A-D asdefined in GOLD 2017, and we collapsed the A-D groups into binary subgroups i.e. “low” comprising groups A and B, and “high” comprising groups C and D [15]. Predicted values of FEV_1_ and FVC were taken from the Global Lung Initiative (GLI) [17]. To quantify the burden from cardiovascular comorbidities we defined a cardiovascular comorbidity count (range 0–5) by summing up the diagnoses of hypertension, coronary artery disease, myocardial infarction, heart failure and heart rhythm disorder.

### Statistical analysis

Data are presented in the tables as numbers or mean values and standard deviations (SD). Comparisons between the three groups (no diagnosis of hyperuricemia, diagnosis of hyperuricemia and disease-specific medication, diagnosis of hyperuricemia and no disease-specific medication) were performed by univariate analysis of variance (ANOVA), or by chi-square-tests in the case of categorical variables. The relationships between variables were assessed using multiple linear regression analysis in case of continuous outcome variables, and binary logistic regression analysis for the exacerbation variable. The distributions of packyears and creatinine were right-skewed and therefore transformed to reduce a potential bias in the numerical estimates of associations. Approximately symmetric distributions were achieved by taking the square root of the variable “packyears”.

The results demonstrated multiple relationships between the variables that could only partially be addressed by conventional regression analysis. There are statistical methods to describe complex networks and especially to differentiate between direct and indirect (i.e. mediated) effects. A common approach is path analysis [18] which has recently been used in COPD research to deal with such situations [19, 20]. Path analysis is particularly capable of integrating the results of a whole set of regression analyses and eliminating non-causal associations. As the procedure is primarily a method to exclude models that do not adequately describe the data [18], the specification of the model typically requires input from pathophysiological knowledge. The resulting model represents a specific hypothesis and is then tested by statistical means. Based on pathophysiological considerations and the results of the regression analyses we constructed a path analysis model, integrating the dependence of variables on risk factors with their dependence on the biomarker UA and then additionally incorporating the relationship to the exacerbation variable. For computation the software package AMOS (IBM Corp., Armonk, NY, USA) with generalized least squares estimation (GLS) was used, and the goodness of fit was described by the chi-square statistics, the comparative fit index (CFI) and the root mean square error of approximation (RMSEA). CFI values ≥0.95 and RMSEA values ≤0.05 are conventionally as indicating a good fit. The chi-square statistics describes the deviation from the model and is therefore acceptable if *p* ≥ 0.05. All other statistical analyses were performed by the package SPSS version 24 (IBM Corp., Armonk, NY, USA). *P*-values < 0.05 were considered as statistically significant.

## Results

### Description of the study population

**Fig. 1:**
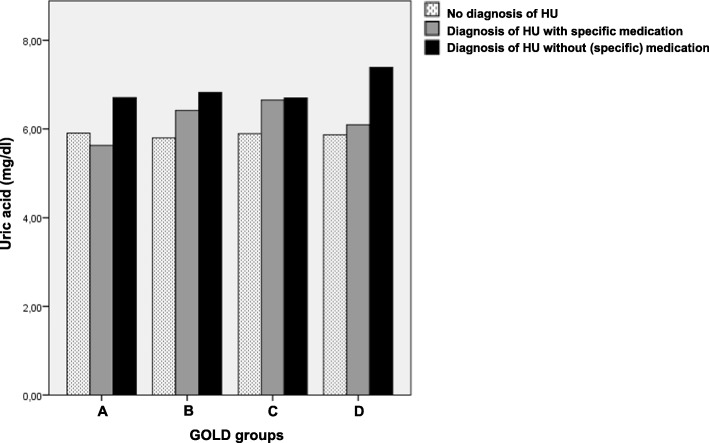
Uric acid levels stratified according to GOLD groups A-D based on the COPD Assessment Test (CAT).The figure shows the uric acid levels for patients without the diagnosis of hyperuricemia, those with a reported diagnosis plus hyperuricemia-specific medication, and those with a reported diagnosis but no or non-specific medication stratified according to GOLD groups A-D. Number of patients in the different GOLD groups: A = 218, B = 1041, C = 32, D = 675

The characteristics of the study population stratified according to the presence or absence of diagnosis and specific treatment are given in Table 1, demonstrating that the levels of uric acid depended on the presence of specific medication in patients with the diagnosis of hyperuricemia. There were significant differences in the serum concentration of UA between the three groups (*p* < 0.001, ANOVA). Post-hoc tests revealed pairwise significant differences between patients without the diagnosis of hyperuricemia, those with specific therapy, and those with diagnosis of HU without specific therapy (*p* < 0.05 each). This underlines that the diagnosis of hyperuricemia is not congruent with elevation of the biomarker uric acid. In addition we stratified UA levels according to the GOLD A-D groups (Fig. 1). The mean number (SD) of cardiovascular comorbidities was 0.96 (±0.99).

**Table 1:** Baseline characteristics of the subgroups

Parameter	Non-HU	HU-specific medication	HU-diagnosis w/o specific medication	*P*-values
N (%)	1610 (81.9%)	174 (8.6%)	182 (9.3%)	–
Gender (m/f)	937/673	154/20	133/49	p < 0.001*
Age (y)	64.2 ± 8.5 [63.8;64.6]	67.7 ± 8.3 [66.7;68.8]	66.3 ± 6.9 [65.3;67.3]	p < 0.001*
BMI (kg/m^2^)	26.2 ± 5.0 [26.0;26.4]	29.6 ± 5.8 [28.8;30.5]	28.4 ± 5.1 [27.7;29.1]	p < 0.001*
Packyears	47.3 ± 34.8 [45.6;49.0]	53.0 ± 31.8 [48.3;57.8]	61.6 ± 44.5 [55.1;68.1]	p < 0.001*
FEV_1_%predicted	52.9 ± 18.6 [52.0;53.8]	53.4 ± 17.8 [50.8;56.1]	53.5 ± 16.6 [51.1;55.9]	*p* = 0.886
FVC%predicted	79.5 ± 18.9 [78.6;80.4]	76.2 ± 18.2 [73.4;78.9]	78.0 ± 18.1 [75.3;80.6]	*p* = 0.061
6-MWD (m)	420.6 ± 106.4 [415.4;425.8]	394.0 ± 102.2 [378.7;409.3]	401.5 ± 100.3 [386.8;416.2]	*p* = 0.001*
Uric acid (mg/dl)	5.83 ± 1.60 [5.75;5.91]	6.22 ± 1.43 [6.00;6.43]	7.02 ± 1.78 [6.76;7.28]	p < 0.001*
GOLD 1/2/3/4	151/685/610/164	16/79/65/14	12/84/76/10	*p* = 0.319
GOLD A/B/C/D	188/859/25/538	15/88/2/69	15/94/5/68	*p* = 0.312
Exa-cat^a^ (low/high)	1047/563	103/71	109/73	*p* = 0.148

The table shows mean values and standard deviations or absolute numbers, as well as 95% confidence intervals in square brackets. Column 4 shows the p-values of comparisons between patients without the diagnosis of hyperuricemia, those with a diagnosis plus hyperuricemia-specific medication, and those with a diagnosis but no hyperuricemia-specific medication (univariate ANOVA or chi-square-tests in the case of categorical variables). Significant (*p* < 0.05) differences are marked with (*). ^a^Exa-cat indicates the exacerbation category as used in the GOLD 2017 ABCD grouping, i.e. “low” comprising the groups A and B, and “high” comprising the groups C and D

### Relationship between variables

The transformed values of packyears (see methods) were used for all subsequent analyses. To properly describe the associations between variables in the presence of multiple correlations we followed a stepwise approach, using linear and logistic regression analyses which were performed using the complete data set (*n* = 1966).

#### Dependence of functional parameters on risk factors

First we determined the associations with the risk factors age, BMI, gender and packyears which were taken as predictors in multiple linear regression analyses. FEV_1_% predicted was dependent on gender, age and BMI (*p* < 0.05 each); FVC % predicted on age (*p* = 0.010); 6-MWD on gender, age and BMI (*p* < 0.05 each); creatinine on gender, age, BMI and packyears (*p* < 0.05 each); and UA on gender, age, BMI and packyears (*p* < 0.05 each). When FEV_1_% predicted was introduced as an additional predictor of 6-MWD, this association was also significant (*p* < 0.001). The count of cardiovascular comorbidities depended on gender, age and BMI (*p* < 0.001 each) but not packyears. Moreover, a logistic regression analysis revealed that the exacerbation category depended, among risk factors, only on age (*p* = 0.005).

#### Associations with uric acid

Second, we determined the associations with UA as predictor of lung function and physical capacity in linear regression analyses, taking into account the risk factors. FEV_1_% predicted and FVC % predicted were dependent on UA (*p* ≤ 0.003), as well as 6-MWD on UA (*p* < 0.001). In addition, the cardiovascular comorbidity count was associated with UA (*p* < 0.001), as well as the binary exacerbation category (*p* < 0.002; logistic regression analysis). Gender was significant (*p* < 0.05 each) in all of these analyses except for FEV_1_% predicted.

#### Exacerbation category versus function and cardiovascular comorbidities

Third, the association between the binary exacerbation category and the functional parameters was analysed using multiple logistic regression, again with gender as covariate. FEV_1_% predicted, 6-MWD (*p* < 0.001 each) and cardiovascular comorbidities (*p* = 0.023) were significant predictors, whereas FVC % predicted was far from significance (*p* = 0.592). The same result was obtained using the cardiovascular comorbidity count as depended variable (*p* < 0.001 each).

#### Integration of results into a path analysis model

The results described above revealed multiple relationships between risk factors, functional variables, exacerbations and biomarkers. To delineate this network of interdependences in greater detail we used the approach of path analysis.

FVC was omitted from these analyses as it was highly correlated with FEV_1_, posing a problem due to collinearity. Moreover, the effects of gender were taken into account by using gender-adjusted values for all variables; the only exception was the binary exacerbation category for which no meaningful adjustment could be introduced. Therefore gender was implicit in the path analyses and disappeared as an explicit predictor. In designing the model we used the results of the regression analyses as well as pathophysiological considerations.

#### Path analysis model

The final model integrated risk factors, UA, functional indices, cardiovascular comorbidities and exacerbations into a comprehensive network that was compatible with the results of the conventional regression analyses. In a first step we used the results of the regression analyses that can be found under “Dependence of functional parameters on risk factors”. Additionally, we introduced correlations between the predictors BMI and packyears, and age and packyears, as these were suggested by the data. In a second step we used the result given under “Associations with uric acid”, to describe the additional relationships between UA, FEV_1_% predicted and 6-MWD. In the third step we incorporated results for the binary exacerbation category and the count of cardiovascular comorbidities as given in “Exacerbation category versus function and cardiovascular comorbidities”. Only associations identified as statistically significant were kept in the final model. It should be noted, that even in the presence of multiple other pathways UA had independent effects on FEV_1_ (*p* = 0.005), 6-MWD (*p* < 0.001) and cardiovascular comorbidity count (*p* < 0.001), whereby UA levels were influenced by age, BMI and packyears. Except for one link (between UA and exacerbation category) all relationships identified as statistically significant in the regression analyses remained significant in the comprehensive model. This consistency, together with the good fit of the data, indicated the validity of the model which we found. The chi-square value of fit with generalized least squares estimation was 12.306 with 8 degrees of freedom and a *p*-value of 0.138, the corresponding CFI 0.996, and the RMSEA 0.017. The standardized regression and correlation coefficients of the final path analysis model are shown in Table 2.

**Table 2:** Results of the final path analysis model

Regression			Estimate	S.E.	C.R.	Standardized	P
Uric acid	←	BMI	.079	.006	12.631	0.273	*p* < 0.001
Uric acid	←	Packyears	.052	.013	4.058	0.088	p < 0.001
Uric acid	←	Age	.019	.004	4.903	0.105	p < 0.001
CV comorbidity	←	Uric acid	.054	.015	3.674	0.083	p < 0.001
CV comorbidity	←	Age	.024	.003	9.235	0.201	p < 0.001
CV comorbidity	←	BMI	.033	.004	7.722	0.173	p < 0.001
FEV_1_	←	Age	.306	.051	6.009	0.137	p < 0.001
FEV_1_	←	Uric acid	−0.790	.284	−2.781	−0.065	0.005
FEV_1_	←	BMI	.565	.083	6.813	0.160	p < 0.001
FEV_1_	←	CV comorbidity	−1.294	.437	−2.963	−0.069	0.003
Exacerbations	←	FEV_1_	−.006	.001	−10.937	−0.240	p < 0.001
Exacerbations	←	CV comorbidity	.033	.011	3.067	0.067	0.002
6-MWD	←	Uric acid	−6.090	1.376	−4.425	−0.087	p < 0.001
6-MWD	←	FEV_1_	2.573	.113	22.856	0.448	p < 0.001
6-MWD	←	Age	−3.025	.249	−12.168	−0.236	p < 0.001
6-MWD	←	BMI	−3.816	.406	−9.402	−0.187	p < 0.001
6-MWD	←	Exacerbations	−22.530	4.276	−5.268	−0.102	p < 0.001
6-MWD	←	CV comorbidity	−7.578	2.120	−3.575	−0.070	p < 0.001
Covariances			Estimate	S.E.	C.R.	Standardized	P
Age	↔	Packyears	1.152	.475	2.428	0.055	*p* < 0.015
BMI	↔	Packyears	1.539	.302	5.102	0.116	p < 0.001

**Fig. 2:**
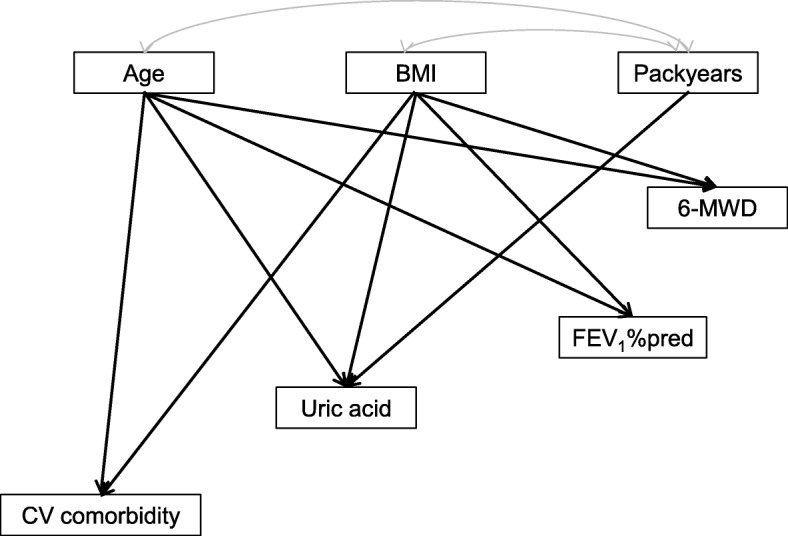
Dependence on risk factors. All variables were adjusted for gender, except for exacerbations and all shown relationships were statistically significant (*p* < 0.05 each). The arched arrows indicate the correlations between predictors. The error terms of the dependent variables have been omitted for the sake of clarity

**Fig. 3:**
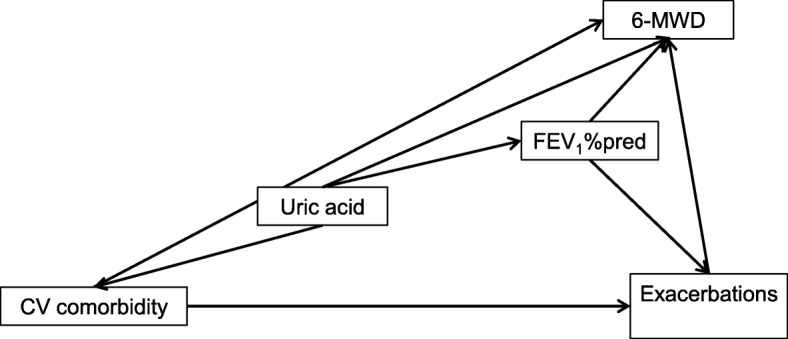
Relationship between all other variables except risk factors. All variables were adjusted for gender, except for exacerbations and all shown relationships were statistically significant (*p* < 0.05 each). The arched arrows indicate the correlations between predictors. The error terms of the dependent variables have been omitted for the sake of clarity

The upper panel refers to the directed arrows (regression terms) depicted in Figs. 2 and 3, whereby the left part lists the arrows shown in these figures. The right part shows the results of the corresponding statistical tests. The first column of the right part shows the non-standardized estimate of the respective regression coefficient, the second column the standard error (S.E.) of this coefficient, the third column the ratio of these two values (critical ratio, C.R.) which is used for significance testing. The forth column shows the standardized estimates of the regression coefficients shown in the first column. The last column shows the significance level based on the generalized least squares (GLS) procedure of AMOS. In an analogous manner the lower panel shows the covariances (bidirectional arrows in Figs 2 and 3) between risk factors, as well as the respective standard errors, critical ratios, correlation coefficients and significance levels

For the purpose of illustration the final model is shown in two parts, the first describing the relationships to risk factors (Fig. 2), and the second the relationship between all other variables except risk factors (Fig. 3). Thus the final model is the overlay of both figures (see Table 2).

### Sensitivity analyses

All analyses described above were performed in the total population of patients (*n* = 1966). As indicated in Table 1, there were significant differences in UA levels between patients with hyperuricemia with vs without specific medication. As these groups were too small to be studied separately in the multivariate analyses we addressed their influence by excluding these subgroups. First, we repeated the path analysis for patients without any diagnosis of hyperuricemia (*n* = 1610); the model of Figs. 2 and 3 was fully confirmed. As the presence of specific medication might affect the associations with UA we then repeated the path analysis excluding the respective group, while keeping that without the diagnosis of hyperuricemia and that with the diagnosis but without specific medication who showed elevated levels of UA (Fig. 1). This resulted in 1792 patients. The path analysis model of Figs. 2 and 3 was fully confirmed, but a significant additional link from UA to exacerbations emerged (*p* = 0.028), underlining the importance of UA per se. This link had been only at the border of statistical significance (*p* = 0.063) when using the whole study population.

## Discussion

Previous studies have indicated a role of uric acid (UA) for mortality, exacerbations and lung function in COPD [1, 2]. The results of our cross-sectional analysis are in line with the different findings and more closely identify its role within the network of functional parameters, exacerbations, cardiovascular comorbidities and risk factors. Beyond conventional regression analyses we used the approach of path analysis to account for both direct and indirect effects of UA as closely as possible. Higher levels UA were linked to higher airway obstruction in terms of FEV_1_, lower physical capacity in terms of 6-MWD and a greater number of cardiovascular comorbidities. Both FEV_1_ and 6-MWD mediated an indirect effect of UA on exacerbations as defined by GOLD 2017. In the absence of hyperuricemia-specific medication there was even a direct link from UA to exacerbations, probably due to the fact that untreated patients had higher uric acid levels (see Fig. 1). Overall, our results were not critically dependent on the inclusion of patients with the diagnosis of hyperuricemia or the presence of specific mediation. The major role was played by UA, strongly suggesting a causative role of UA itself for clinically relevant outcomes in COPD.

The observed association of lung function with UA is in line with previous results regarding FVC and FEV_1_ in lung healthy subjects [9], or FEV_1_ in COPD patients [2]. In our study population we found both FVC and FEV_1_ to be linked to UA. In parallel to findings in patients with pulmonary hypertension [21], we observed that 6-MWD was also linked to UA in patients with COPD. Moreover, UA levels have been shown to be related to exacerbations in patients with COPD [2], as well as cardiovascular comorbidities [22]. Both findings were confirmed by our data which therefore are fully compatible with the known link between UA and mortality [1].

UA levels are known to be influenced by a variety of factors, among them overweight, age and male gender. These risk factors were also significant predictors in our data. In the path analysis gender was implicit and taken into account by the use of appropriately adjusted values. As excretion rate has an effect on UA levels [9], we tentatively also included creatinine as a biomarker in additional analyses. Importantly, this did not lead to a change of the role played by UA in our network. Higher values of BMI were associated with higher values of FEV_1_, which is a common finding in COPD [19]. At the same time, there were associated with higher levels of UA, however UA itself had a negative effect on FEV_1_, pointing towards greater airflow limitation. Taken together, this observation again underlines that UA itself has effects that are explained by common risk factors like BMI.

Thus, patients with hyperuricemia showed different ranges of UA depending on the presence of specific medication as illustrated in Fig. 1. The associations identified by path analysis became even stronger when excluding patients with specific medication. Remarkably, most of the previous studies investigated the role of UA in COPD irrespective of a diagnosis of hyperuricemia. As there were no major differences when including or excluding these patients, our data support this approach. The most important factor seemed to be the level of UA not the diagnosis. On the other hand, our observations regarding the additional link between UA and exacerbations indicate that the presence of hyperuricemia-specific medication can modulate the results, suggesting that future analyses should take account of diagnosis, medication and biomarker. As a secondary finding, Fig. 1 demonstrates the effectiveness of hyperuricemia-specific therapy in our study population but also shows that UA levels were still higher than in patients without the diagnosis of hyperuricemia.

Beyond lung function and physical capacity, the rate and severity of exacerbations are determinants of the prognosis in COPD [12, 23]. We coded exacerbations through a binary variable equivalent to the difference between the AB and CD groups in the recent GOLD recommendations [15]. This was motivated by the aim to use a definition close to that established for therapeutical decisions. Exacerbations defined in this way were dependent on lung function and by themselves had a negative effect on 6-MWD, in accordance with previous data [24, 25]. The link to 6-MWD appears plausible since exacerbations often lead to an irreversible deterioration of clinical state. Despite the indirect effects of UA mediated via both exacerbations and FEV_1_, it was also directly associated with 6-MWD. We do not know whether this could be partially due persistent motoric impairment from gout arthritis even in the absence of acute episodes.

In the path analysis model exacerbations were dependent on FEV_1_. The independent effect of UA on exacerbations was statistically significant only when excluding patients with hyperuricemia-specific medication. This seems understandable due to the reduction in the range of variation of UA compared to patients with hyperuricemia without specific UA-lowering medication. These observations support the hypothesis that UA influences the risk/severity of COPD exacerbations. Taking into account the different paths linking UA to exacerbations, our findings suggested that an increase in UA level by 2 mg/dl was linked to a shift by about 5% from the low to the high exacerbation category. A potential pathophysiological link could be endothelin-1 which is known to be elevated in hyperuricemia, but also in asthma and COPD exacerbations [5]. Furthermore, pro-inflammatory effects of UA in terms of TNF-alpha activation have been described [26]. The associations of the cardiovascular comorbidity count with UA but also functional measures and exacerbations suggest that UA triggers pro-inflammatory processes that are relevant for cardiovascular diseases [22] which in turn have an impact on COPD prognosis [27]. Taken together, it might well be that UA exerts part of its effects on functional state and mortality of COPD via a set of inflammatory compounds.

### Limitations and strength

Obvious limitations of the study are its cross-sectional and non-interventional design. Furthermore, we did not have data on the history of clinical manifestations of gout, in terms of acute episodes. Patients with the physician-based diagnosis of hyperuricemia are potentially more health-conscious and may therefore have a better physical capacity and prognosis, which could influence our results. Moreover, there was no information on the specific medical reasons why patients received hyperuricemia-specific treatment. Also, there was no information on the duration of the hyperuricemia-specific treatment. This, however, appeared to be of minor importance, since the major results were independent of excluding or including these patients. Long-term follow-up data could reveal whether the relationships observed in a cross-sectional analysis are also reflected in the relationships between the changes of parameters within the follow-up period. The strength of our study was its large sample size which allowed the use of sophisticated statistical methods with the aim to disentangle the role of UA from other influencing factors. Moreover we could rely on comprehensive, high quality data of lung function and patients’ clinical characteristics.

## Conclusion

Within the limits of a cross-sectional approach, our results strongly suggest that uric acid is not only a useful biomarker for single entities in COPD but also plays a central role in this disease. It is simultaneously associated with determinants of worse COPD prognosis including airway obstruction, cardiovascular comorbidities, exacerbations and physical capacity, irrespective of common risk factors. This suggests that more attention should be paid to the levels of uric acid in the evaluation of COPD disease status. The described role of uric acid should be substantiated in future targeted intervention trials.

## Abbreviations

6-MWD: 6-min walk distance
BMI: Body mass index
CAT: COPD Assessment Test
CFI: Comparative fit index
COPD: Chronic obstructive pulmonary disease
FEV_1_: Forced expiratory volume in 1 s
FVC: Forced vital capacity
GLI: Global Lung Initiative
GLS: Generalized least squares estimation
RMSEA: Root mean square error of approximation (RMSEA)
SD: Standard deviations
UA: Uric acid
